# [*N*′-(3-Eth­oxy-2-oxidobenzyl­idene)-4-hy­droxy-3-meth­oxy­benzohydrazidato](methanol)dioxidomolybdenum(VI)

**DOI:** 10.1107/S1600536812008549

**Published:** 2012-03-03

**Authors:** Shou-Xing Wang

**Affiliations:** a Laboratory Management Center, Zaozhuang University, Zaozhuang 277160, People’s Republic of China

## Abstract

In the title dioxidomolybdenum(VI) complex, [Mo(C_17_H_16_N_2_O_5_)O_2_(CH_3_OH)], the Mo^VI^ atom is coordinated by the phenolate O, imine N and enolic O atoms of the tridentate hydrazone ligand, one methanol O atom, and two oxide O atoms, forming a distorted octa­hedral coordination geometry. The oxide O atoms adopt a *cis* conformation: one is *trans* to the methanol O atom and the other is *trans* to the ligand N atom. The dihedral angle between the two benzene rings in the hydrazone ligand is 4.0 (3)°. In the crystal, mol­ecules are linked by O—H⋯N and O—H⋯O hydrogen bonds.

## Related literature

For background to molybdenum complexes with hydrazone ligands, see: Dinda *et al.* (2003 ▶); Vrdoljak *et al.* (2005 ▶); Debel *et al.* (2008 ▶). For similar complexes, see: Sheikhshoaie *et al.* (2011 ▶); Gao *et al.* (2004 ▶); Saeednia *et al.* (2009 ▶).
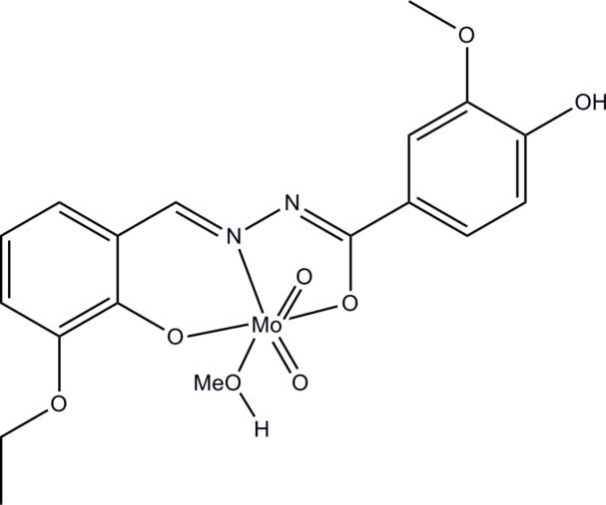


## Experimental

### 

#### Crystal data

[Mo(C_17_H_16_N_2_O_5_)O_2_(CH_4_O)]
*M* *_r_* = 488.30
Monoclinic, 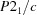

*a* = 10.054 (2) Å
*b* = 16.401 (3) Å
*c* = 12.233 (3) Å
β = 101.946 (2)°
*V* = 1973.5 (7) Å^3^
*Z* = 4
Mo *K*α radiation
μ = 0.71 mm^−1^
*T* = 298 K
0.23 × 0.21 × 0.20 mm

#### Data collection

Bruker SMART CCD diffractometer
Absorption correction: multi-scan (*SADABS*; Sheldrick, 1996 ▶) *T*_min_ = 0.853, *T*_max_ = 0.871
11140 measured reflections
4300 independent reflections
3162 reflections with *I* > 2σ(*I*)
*R*_int_ = 0.040

#### Refinement

*R*[*F* ^2^ > 2σ(*F* ^2^)] = 0.044
*wR*(*F* ^2^) = 0.093
*S* = 1.03
4300 reflections
269 parameters
1 restraint
H atoms treated by a mixture of independent and constrained refinement
Δρ_max_ = 0.62 e Å^−3^
Δρ_min_ = −0.69 e Å^−3^

### 

Data collection: *SMART* (Bruker, 1998 ▶); cell refinement: *SAINT* (Bruker, 1998 ▶); data reduction: *SAINT*; program(s) used to solve structure: *SHELXS97* (Sheldrick, 2008 ▶); program(s) used to refine structure: *SHELXL97* (Sheldrick, 2008 ▶); molecular graphics: *SHELXTL* (Sheldrick, 2008 ▶); software used to prepare material for publication: *SHELXTL*.

## Supplementary Material

Crystal structure: contains datablock(s) global, I. DOI: 10.1107/S1600536812008549/hb6656sup1.cif

Structure factors: contains datablock(s) I. DOI: 10.1107/S1600536812008549/hb6656Isup2.hkl

Additional supplementary materials:  crystallographic information; 3D view; checkCIF report

## Figures and Tables

**Table 1:** Selected bond lengths (Å)

Mo1—O8	1.683 (3)
Mo1—O7	1.707 (2)
Mo1—O1	1.920 (2)
Mo1—O3	2.009 (2)
Mo1—N1	2.238 (3)
Mo1—O6	2.364 (3)

**Table 2:** Hydrogen-bond geometry (Å, °)

*D*—H⋯*A*	*D*—H	H⋯*A*	*D*⋯*A*	*D*—H⋯*A*
O6—H6⋯N2^i^	0.85 (1)	2.01 (1)	2.853 (4)	175 (5)
O5—H5⋯O4	0.82	2.20	2.646 (4)	114
O5—H5⋯O7^ii^	0.82	2.12	2.828 (3)	145

Symmetry codes: (i) 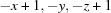; (ii) 
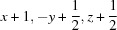
.

**Fig. 1.**
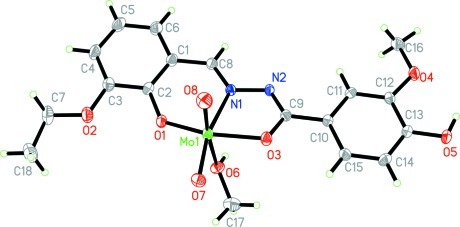
The molecular structure of the title complex, showing displacement ellipsoids drawn at the 30% probability level.

**Fig. 2.**
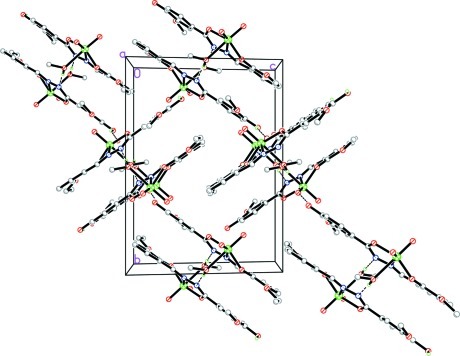
The molecular packing structure of the title complex, viewed along the a axis. Hydrogen bonds are drawn as dashed lines.

## supplementary crystallographic information

### Comment

Molybdenum complexes with hydrazones have received much attention for their structures and catalytic properties (Dinda *et al.*, 2003; Vrdoljak *et al.*, 2005; Debel *et al.*, 2008). In the present work, the author reports the title new dioxomolybdenum(VI) complex with a new hydrazone ligand *N'*-[(3-ethoxy-2-hydroxybenzylidene]-4-hydroxy-3- methoxybenzohydrazide.

In the title complex, Fig. 1, the Mo atom is six-coordinated by the phenolate O, imine N, and enolic O atoms of the hydrazone ligand, one methanol O atom, and two oxide O atoms, forming an octahedral geometry. The dihedral angle between the two benzene rings in the hydrazone ligand is 4.0 (3)°. The lengths of Mo—O and Mo—N bonds (Table 1) are within normal values (Sheikhshoaie *et al.*, 2011; Gao *et al.*, 2004; Saeednia *et al.*, 2009). The crystal of the complex features intermolecular O—H···N and O—H···O hydrogen bonds (Table 2, Fig. 2).

### Experimental

The title compound was obtained by stirring 3-ethoxysalicylaldehyde (0.1 mmol, 16.6 mg), 4-hydroxy-3-methoxybenzohydrazide (0.1 mmol, 18.2 mg), and MoO_2_(acac)_2_ (0.1 mmol, 32.6 mg) in methanol (20 ml) for 30 min. The reaction mixture was then filtered. Yellow block-shaped single crystals were formed from the filtrate after a week.

### Refinement

The methanol H atom was located from a difference Fourier map and refined isotropically, with O—H distance restrained to 0.85 (1) Å. The remaining hydrogen atoms were placed in geometrically idealized positions and constrained to ride on their parent atoms, with C—H distances in the range 0.93–0.97 Å, O—H distance of 0.82 Å, and with *U*_iso_(H) set at 1.2 or 1.5*U*_eq_(C, O).

### Crystal data

**Table:**

[Mo(C_17_H_16_N_2_O_5_)O_2_(CH_4_O)]	*F*(000) = 992
*M**_r_* = 488.30	*D*_x_ = 1.643 Mg m^−^^3^
Monoclinic, *P*2_1_/*c*	Mo *K*α radiation, λ = 0.71073 Å
*a* = 10.054 (2) Å	Cell parameters from 2809 reflections
*b* = 16.401 (3) Å	θ = 2.7–25.1°
*c* = 12.233 (3) Å	µ = 0.71 mm^−^^1^
β = 101.946 (2)°	*T* = 298 K
*V* = 1973.5 (7) Å^3^	Block, yellow
*Z* = 4	0.23 × 0.21 × 0.20 mm

### Data collection

**Table:**

Bruker SMART CCD diffractometer	4300 independent reflections
Radiation source: fine-focus sealed tube	3162 reflections with *I* > 2σ(*I*)
Graphite monochromator	*R*_int_ = 0.040
ω scan	θ_max_ = 27.0°, θ_min_ = 2.1°
Absorption correction: multi-scan (*SADABS*; Sheldrick, 1996)	*h* = −12→10
*T*_min_ = 0.853, *T*_max_ = 0.871	*k* = −13→20
11140 measured reflections	*l* = −15→15

### Refinement

**Table:**

Refinement on *F*^2^	Primary atom site location: structure-invariant direct methods
Least-squares matrix: full	Secondary atom site location: difference Fourier map
*R*[*F*^2^ > 2σ(*F*^2^)] = 0.044	Hydrogen site location: inferred from neighbouring sites
*wR*(*F*^2^) = 0.093	H atoms treated by a mixture of independent and constrained refinement
*S* = 1.03	*w* = 1/[σ^2^(*F*_o_^2^) + (0.0346*P*)^2^ + 1.5131*P*] where *P* = (*F*_o_^2^ + 2*F*_c_^2^)/3
4300 reflections	(Δ/σ)_max_ < 0.001
269 parameters	Δρ_max_ = 0.62 e Å^−^^3^
1 restraint	Δρ_min_ = −0.69 e Å^−^^3^

### Special details

**Table:**

Geometry. All e.s.d.'s (except the e.s.d. in the dihedral angle between two l.s. planes) are estimated using the full covariance matrix. The cell e.s.d.'s are taken into account individually in the estimation of e.s.d.'s in distances, angles and torsion angles; correlations between e.s.d.'s in cell parameters are only used when they are defined by crystal symmetry. An approximate (isotropic) treatment of cell e.s.d.'s is used for estimating e.s.d.'s involving l.s. planes.
Refinement. Refinement of *F*^2^ against ALL reflections. The weighted *R*-factor *wR* and goodness of fit *S* are based on *F*^2^, conventional *R*-factors *R* are based on *F*, with *F* set to zero for negative *F*^2^. The threshold expression of *F*^2^ > σ(*F*^2^) is used only for calculating *R*-factors(gt) *etc*. and is not relevant to the choice of reflections for refinement. *R*-factors based on *F*^2^ are statistically about twice as large as those based on *F*, and *R*- factors based on ALL data will be even larger.

### Fractional atomic coordinates and isotropic or equivalent isotropic displacement parameters (Å^2^)

**Table:**

	*x*	*y*	*z*	*U*_iso_*/*U*_eq_	
Mo1	0.20815 (3)	0.10239 (2)	0.35081 (2)	0.02894 (11)	
N1	0.4173 (3)	0.05321 (18)	0.3574 (2)	0.0250 (7)	
N2	0.5200 (3)	0.08381 (18)	0.4423 (2)	0.0260 (7)	
O1	0.1607 (2)	0.00584 (16)	0.2626 (2)	0.0354 (6)	
O2	−0.0005 (3)	−0.07464 (17)	0.1042 (2)	0.0449 (7)	
O3	0.3379 (2)	0.15737 (15)	0.4761 (2)	0.0332 (6)	
O4	0.9131 (2)	0.23736 (17)	0.7064 (2)	0.0410 (7)	
O5	0.7908 (3)	0.3165 (2)	0.8463 (2)	0.0507 (8)	
H5	0.8685	0.3204	0.8357	0.076*	
O6	0.2310 (3)	0.00410 (17)	0.4948 (2)	0.0357 (6)	
O7	0.0629 (2)	0.12772 (16)	0.3944 (2)	0.0381 (7)	
O8	0.2216 (3)	0.16890 (18)	0.2489 (2)	0.0459 (7)	
C1	0.3597 (4)	−0.0307 (2)	0.1922 (3)	0.0337 (9)	
C2	0.2189 (4)	−0.0313 (2)	0.1862 (3)	0.0307 (9)	
C3	0.1332 (4)	−0.0746 (2)	0.0993 (3)	0.0355 (9)	
C4	0.1891 (4)	−0.1132 (3)	0.0195 (3)	0.0460 (11)	
H4	0.1330	−0.1412	−0.0384	0.055*	
C5	0.3281 (5)	−0.1108 (3)	0.0245 (3)	0.0519 (12)	
H5A	0.3638	−0.1364	−0.0309	0.062*	
C6	0.4132 (4)	−0.0715 (3)	0.1099 (3)	0.0440 (11)	
H6A	0.5065	−0.0717	0.1134	0.053*	
C7	−0.0893 (4)	−0.1285 (3)	0.0308 (4)	0.0546 (13)	
H7A	−0.0566	−0.1842	0.0420	0.065*	
H7B	−0.0922	−0.1136	−0.0464	0.065*	
C8	0.4533 (4)	0.0069 (2)	0.2837 (3)	0.0318 (9)	
H8	0.5456	−0.0029	0.2898	0.038*	
C9	0.4682 (3)	0.1391 (2)	0.4979 (3)	0.0261 (8)	
C10	0.5546 (3)	0.1847 (2)	0.5889 (3)	0.0251 (8)	
C11	0.6961 (3)	0.1855 (2)	0.6019 (3)	0.0276 (8)	
H11	0.7371	0.1563	0.5527	0.033*	
C12	0.7752 (3)	0.2295 (2)	0.6876 (3)	0.0288 (8)	
C13	0.7136 (4)	0.2728 (2)	0.7621 (3)	0.0347 (9)	
C14	0.5754 (4)	0.2711 (3)	0.7500 (3)	0.0431 (11)	
H14	0.5351	0.2992	0.8007	0.052*	
C15	0.4948 (4)	0.2285 (2)	0.6640 (3)	0.0357 (9)	
H15	0.4008	0.2289	0.6560	0.043*	
C16	0.9849 (4)	0.1843 (3)	0.6466 (4)	0.0459 (11)	
H16A	0.9636	0.1287	0.6605	0.069*	
H16B	1.0809	0.1931	0.6708	0.069*	
H16C	0.9585	0.1955	0.5680	0.069*	
C17	0.1822 (5)	0.0181 (3)	0.5950 (4)	0.0558 (12)	
H17A	0.2257	0.0655	0.6322	0.084*	
H17B	0.2025	−0.0284	0.6433	0.084*	
H17C	0.0857	0.0265	0.5767	0.084*	
C18	−0.2272 (5)	−0.1219 (3)	0.0562 (4)	0.0709 (16)	
H18A	−0.2235	−0.1373	0.1325	0.106*	
H18B	−0.2886	−0.1574	0.0076	0.106*	
H18C	−0.2586	−0.0666	0.0450	0.106*	
H6	0.303 (3)	−0.024 (3)	0.511 (4)	0.080*	

### Atomic displacement parameters (Å^2^)

**Table:**

	*U*^11^	*U*^22^	*U*^33^	*U*^12^	*U*^13^	*U*^23^
Mo1	0.02297 (16)	0.0324 (2)	0.02923 (16)	0.00327 (15)	0.00036 (11)	−0.00289 (16)
N1	0.0226 (14)	0.0258 (18)	0.0255 (14)	0.0006 (13)	0.0022 (11)	−0.0011 (13)
N2	0.0231 (14)	0.0284 (19)	0.0250 (14)	−0.0023 (13)	0.0014 (12)	−0.0044 (13)
O1	0.0278 (13)	0.0443 (17)	0.0327 (13)	−0.0031 (12)	0.0028 (11)	−0.0133 (12)
O2	0.0377 (16)	0.0480 (19)	0.0451 (16)	−0.0068 (13)	0.0001 (13)	−0.0127 (14)
O3	0.0254 (13)	0.0348 (16)	0.0366 (14)	0.0043 (11)	0.0001 (11)	−0.0101 (12)
O4	0.0257 (13)	0.0491 (19)	0.0464 (16)	−0.0047 (13)	0.0033 (12)	−0.0179 (14)
O5	0.0381 (16)	0.067 (2)	0.0477 (17)	−0.0168 (16)	0.0106 (13)	−0.0329 (16)
O6	0.0380 (15)	0.0371 (17)	0.0331 (14)	0.0124 (12)	0.0094 (12)	0.0000 (12)
O7	0.0253 (13)	0.0424 (17)	0.0457 (15)	0.0103 (12)	0.0055 (11)	−0.0024 (13)
O8	0.0429 (16)	0.0486 (19)	0.0431 (16)	0.0050 (14)	0.0017 (13)	0.0102 (14)
C1	0.035 (2)	0.033 (2)	0.0323 (19)	0.0005 (18)	0.0052 (16)	−0.0063 (17)
C2	0.036 (2)	0.030 (2)	0.0248 (18)	0.0018 (17)	0.0026 (15)	−0.0005 (16)
C3	0.039 (2)	0.031 (2)	0.034 (2)	−0.0017 (18)	0.0019 (17)	−0.0013 (17)
C4	0.051 (3)	0.047 (3)	0.038 (2)	−0.003 (2)	0.0029 (19)	−0.017 (2)
C5	0.055 (3)	0.060 (3)	0.042 (2)	0.004 (2)	0.014 (2)	−0.024 (2)
C6	0.036 (2)	0.053 (3)	0.044 (2)	0.004 (2)	0.0085 (18)	−0.015 (2)
C7	0.050 (3)	0.050 (3)	0.055 (3)	−0.007 (2)	−0.011 (2)	−0.014 (2)
C8	0.0244 (18)	0.038 (2)	0.0326 (19)	0.0040 (17)	0.0042 (15)	−0.0066 (18)
C9	0.0266 (18)	0.027 (2)	0.0237 (17)	−0.0015 (16)	0.0028 (14)	0.0021 (16)
C10	0.0284 (18)	0.021 (2)	0.0251 (16)	0.0006 (15)	0.0035 (14)	0.0007 (15)
C11	0.0303 (18)	0.027 (2)	0.0261 (17)	0.0027 (16)	0.0081 (14)	−0.0029 (16)
C12	0.0262 (18)	0.029 (2)	0.0297 (18)	−0.0013 (16)	0.0019 (15)	0.0006 (16)
C13	0.035 (2)	0.039 (3)	0.0302 (19)	−0.0065 (18)	0.0065 (16)	−0.0095 (18)
C14	0.040 (2)	0.050 (3)	0.042 (2)	0.001 (2)	0.0143 (18)	−0.018 (2)
C15	0.0254 (19)	0.040 (3)	0.042 (2)	−0.0021 (17)	0.0084 (16)	−0.0104 (19)
C16	0.031 (2)	0.051 (3)	0.058 (3)	0.001 (2)	0.0139 (19)	−0.009 (2)
C17	0.050 (3)	0.062 (3)	0.059 (3)	0.009 (2)	0.021 (2)	0.007 (3)
C18	0.046 (3)	0.082 (4)	0.078 (4)	−0.014 (3)	−0.001 (3)	−0.011 (3)

### Geometric parameters (Å, º)

**Table:**

Mo1—O8	1.683 (3)	C5—C6	1.367 (5)
Mo1—O7	1.707 (2)	C5—H5A	0.9300
Mo1—O1	1.920 (2)	C6—H6A	0.9300
Mo1—O3	2.009 (2)	C7—C18	1.487 (6)
Mo1—N1	2.238 (3)	C7—H7A	0.9700
Mo1—O6	2.364 (3)	C7—H7B	0.9700
N1—C8	1.286 (4)	C8—H8	0.9300
N1—N2	1.398 (4)	C9—C10	1.467 (4)
N2—C9	1.305 (4)	C10—C15	1.397 (5)
O1—C2	1.347 (4)	C10—C11	1.399 (5)
O2—C3	1.358 (4)	C11—C12	1.380 (5)
O2—C7	1.432 (4)	C11—H11	0.9300
O3—C9	1.316 (4)	C12—C13	1.398 (5)
O4—C12	1.364 (4)	C13—C14	1.366 (5)
O4—C16	1.425 (4)	C14—C15	1.378 (5)
O5—C13	1.360 (4)	C14—H14	0.9300
O5—H5	0.8200	C15—H15	0.9300
O6—C17	1.430 (5)	C16—H16A	0.9600
O6—H6	0.847 (10)	C16—H16B	0.9600
C1—C2	1.402 (5)	C16—H16C	0.9600
C1—C6	1.405 (5)	C17—H17A	0.9600
C1—C8	1.444 (5)	C17—H17B	0.9600
C2—C3	1.413 (5)	C17—H17C	0.9600
C3—C4	1.377 (5)	C18—H18A	0.9600
C4—C5	1.386 (6)	C18—H18B	0.9600
C4—H4	0.9300	C18—H18C	0.9600
			
O8—Mo1—O7	106.19 (13)	C18—C7—H7A	110.1
O8—Mo1—O1	99.61 (12)	O2—C7—H7B	110.1
O7—Mo1—O1	104.31 (12)	C18—C7—H7B	110.1
O8—Mo1—O3	97.79 (12)	H7A—C7—H7B	108.4
O7—Mo1—O3	96.60 (11)	N1—C8—C1	124.3 (3)
O1—Mo1—O3	147.72 (10)	N1—C8—H8	117.9
O8—Mo1—N1	92.37 (12)	C1—C8—H8	117.9
O7—Mo1—N1	159.23 (11)	N2—C9—O3	122.7 (3)
O1—Mo1—N1	81.09 (10)	N2—C9—C10	120.8 (3)
O3—Mo1—N1	71.19 (10)	O3—C9—C10	116.5 (3)
O8—Mo1—O6	169.67 (11)	C15—C10—C11	119.1 (3)
O7—Mo1—O6	83.73 (11)	C15—C10—C9	119.6 (3)
O1—Mo1—O6	80.25 (10)	C11—C10—C9	121.3 (3)
O3—Mo1—O6	77.86 (10)	C12—C11—C10	120.3 (3)
N1—Mo1—O6	77.38 (9)	C12—C11—H11	119.9
C8—N1—N2	117.5 (3)	C10—C11—H11	119.9
C8—N1—Mo1	125.9 (2)	O4—C12—C11	125.7 (3)
N2—N1—Mo1	116.2 (2)	O4—C12—C13	114.5 (3)
C9—N2—N1	108.9 (3)	C11—C12—C13	119.8 (3)
C2—O1—Mo1	132.1 (2)	O5—C13—C14	120.1 (3)
C3—O2—C7	117.9 (3)	O5—C13—C12	120.1 (3)
C9—O3—Mo1	121.0 (2)	C14—C13—C12	119.8 (3)
C12—O4—C16	117.4 (3)	C13—C14—C15	121.1 (4)
C13—O5—H5	109.5	C13—C14—H14	119.4
C17—O6—Mo1	122.1 (2)	C15—C14—H14	119.4
C17—O6—H6	108 (3)	C14—C15—C10	119.9 (3)
Mo1—O6—H6	120 (3)	C14—C15—H15	120.1
C2—C1—C6	119.4 (3)	C10—C15—H15	120.1
C2—C1—C8	122.2 (3)	O4—C16—H16A	109.5
C6—C1—C8	118.3 (3)	O4—C16—H16B	109.5
O1—C2—C1	122.6 (3)	H16A—C16—H16B	109.5
O1—C2—C3	117.7 (3)	O4—C16—H16C	109.5
C1—C2—C3	119.7 (3)	H16A—C16—H16C	109.5
O2—C3—C4	125.7 (3)	H16B—C16—H16C	109.5
O2—C3—C2	115.0 (3)	O6—C17—H17A	109.5
C4—C3—C2	119.3 (4)	O6—C17—H17B	109.5
C3—C4—C5	120.7 (4)	H17A—C17—H17B	109.5
C3—C4—H4	119.6	O6—C17—H17C	109.5
C5—C4—H4	119.6	H17A—C17—H17C	109.5
C6—C5—C4	120.9 (4)	H17B—C17—H17C	109.5
C6—C5—H5A	119.6	C7—C18—H18A	109.5
C4—C5—H5A	119.6	C7—C18—H18B	109.5
C5—C6—C1	120.0 (4)	H18A—C18—H18B	109.5
C5—C6—H6A	120.0	C7—C18—H18C	109.5
C1—C6—H6A	120.0	H18A—C18—H18C	109.5
O2—C7—C18	108.1 (4)	H18B—C18—H18C	109.5
O2—C7—H7A	110.1		

### Hydrogen-bond geometry (Å, º)

**Table:**

*D*—H···*A*	*D*—H	H···*A*	*D*···*A*	*D*—H···*A*
O6—H6···N2^i^	0.85 (1)	2.01 (1)	2.853 (4)	175 (5)
O5—H5···O4	0.82	2.20	2.646 (4)	114
O5—H5···O7^ii^	0.82	2.12	2.828 (3)	145

Symmetry codes: (i) −*x*+1, −*y*, −*z*+1; (ii) *x*+1, −*y*+1/2, *z*+1/2.
